# Calcitonin and Bone Physiology: In Vitro, In Vivo, and Clinical Investigations

**DOI:** 10.1155/2020/3236828

**Published:** 2020-09-10

**Authors:** Jingbo Xie, Jian Guo, Zaeema Kanwal, Mingzheng Wu, Xiangyang Lv, Nihal Abdalla Ibrahim, Ping Li, Manal Ali Buabeid, El-Shaimaa A. Arafa, Qingshan Sun

**Affiliations:** ^1^Department of Orthopedics, Fengcheng People's Hospital, Fengcheng, Jiangxi 331100, China; ^2^Department of the Second Orthopedics, Hongdu Hospital of Traditional Chinese Medicine Affiliated to Jiangxi University of Traditional Chinese Medicine, Nanchang Hongdu Traditional Chinese Medicine Hospital, Nanchang, Jiangxi 330008, China; ^3^Ameer Ud Din Medical College, Lahore, Pakistan; ^4^Department of Orthopaedics, Pu'ai Hospital, Tongji Medical College, Huazhong University of Science and Technology, Wuhan, Hubei 430000, China; ^5^Department of Orthopaedics, Xi'an International Medical Center Hospital, Xi'an, Shaanxi 710100, China; ^6^Department of Clinical Sciences, Ajman University, Ajman 346, UAE; ^7^Department of Orthopaedics, Ya'an People's Hospital, Ya'an, Sichuan 625000, China; ^8^Department of Orthopedics, The Third Hospital of Shandong Province, Jinan, Shandong 250031, China

## Abstract

Calcitonin was discovered as a peptide hormone that was known to reduce the calcium levels in the systemic circulation. This hypocalcemic effect is produced due to multiple reasons such as inhibition of bone resorption or suppression of calcium release from the bone. Thus, calcitonin was said as a primary regulator of the bone resorption process. This is the reason why calcitonin has been used widely in clinics for the treatment of bone disorders such as osteoporosis, hypercalcemia, and Paget's disease. However, presently calcitonin usage is declined due to the development of efficacious formulations of new drugs. Calcitonin gene-related peptides and several other peptides such as intermedin, amylin, and adrenomedullin (ADM) are categorized in calcitonin family. These peptides are known for the structural similarity with calcitonin. Aside from having a similar structure, these peptides have few overlapping biological activities and signal transduction action through related receptors. However, several other activities are also present that are peptide specific. *In vitro* and *in vivo* studies documented the posttreatment effects of calcitonin peptides, i.e., positive effect on bone osteoblasts and their formation and negative effect on osteoclasts and their resorption. The recent research studies carried out on genetically modified mice showed the inhibition of osteoclast activity by amylin, while astonishingly calcitonin plays its role by suppressing osteoblast and bone turnover. This article describes the review of the bone, the activity of the calcitonin family of peptides, and the link between them.

## 1. Introduction

### 1.1. Calcitonin Peptides

When the level of serum calcium is raised due to parafollicular cells present in the thyroid gland, it results in the secretion of peptide calcitonin. Calcitonin is a peptide in nature. Different types of peptide hormones respond differently towards signals and express in different tissues. These hormones show structural analogy with calcitonin and transduce signals via various receptors. Apart from having a similar structure, some of the peptides have few overlapping biological activities, while others are unique.

#### 1.1.1. Calcitonin in Homeostasis

The serum calcium level is maintained within a narrow range of 8.5 mg/dL and 10.5 mg/dL by the coordinated actions of the skeleton, the gut, and the kidneys. The regulation of calcium level is necessary because it plays critical role in many essential physiological processes such as coagulation, contraction of muscle, and glycogenolysis. Similarly, Ca^++^ controls cellular adhesions [1]. Physicians have already recognized the significance of the regulation of calcium level and the detrimental outcome of the disproportional level of calcium. In the late 19^th^ century, the parathyroid gland was discovered that lead to the empathy of calcium homeostasis and hormonal mechanisms [2]. Later, in 1925, the researchers [3] described the physiological function of the parathyroid gland by showing that tetany caused by parathyroidectomy was treated by the acid extracts of the parathyroid gland. The researchers revealed that, at a low level of calcium, this gland secretes parathyroid hormone (PTH) to restore the calcium level to its standard range. The investigations should be carried out to explore the mechanism of action of PTH by the advanced improvement of PTH extraction methods [4, 5]. Another group of researchers investigated the regulation of calcium levels by thyroid-parathyroid gland and calcium homeostasis in the perfusion system of anaesthetized dogs [6]. At high levels of calcium, perfusion of thyroid-parathyroid glands causes a rapid decrease in blood calcium level within fifteen minutes. According to a hypothesis, the permeation of high amount of calcium inhibits the excretion of PTH that causes a rapid fall in systemic calcium levels, as PTH is the only hormone released from the parathyroid gland. The investigators performed a test on dogs by removing their thyroid-parathyroid glands to confirm the hypothesis as it was predicted that similar effects could be seen on the calcium level in the body in PTH-free environment. Surprisingly, the systemic calcium level was maintained at a high level. However, it was concluded that hypercalcemia causes the production of a hormone which reduces the blood calcium level and does not inhibit the production of PTH [6, 7]. The hormone was named as “calcitonin” since it controls the calcium tone. Many studies are carried out that suggest another name of calcitonin as “thyrocalcitonin” since calcitonin is formed by the thyroid gland [8]. The biological assay is used to study calcitonin by determining methodology of parathyroid hormone by the regulation of calcium level in the body. A previous study [9] involved the injection of ^45^Ca to pregnant rats and incorporated the embryonic bone in the tissue culture. This bioassay was needed to evaluate the bone resorption and also the release of radioactivity-labeled calcium. Bone resorption was induced under the effect of calcitonin that was purified mildly and extracted from the thyroid gland of the rat. Thus, the bone resorption process was determined in grouped format with PTH only in baseline conditions. These results identified the hypocalcemic mechanism of calcitonin and resulted in a decreased level of calcitonin at both PTH enthused and basal bone resorption [9].

The clinical trial conducted *in vivo* estimated the hydroxyproline calcitonin produced by collagen breakdown and showed further evidence of calcitonin-based inhibition of bone resorption [10]. The result showed that calcitonin directly suppressed the bone resorption and collagen breakdown as it rapidly reduced the excretion of urinary hydroxyproline. Calcitonin was purified from various animal species, including the mammals, the fishes, and the birds [11]. In the human thyroid gland, calcitonin is produced in a large quantity that is why purification of calcitonin was proved to be quiet challenging. Neher et al. [12] purified human calcitonin (hCT) from the patients having tumors of the thyroid C cells, which forms calcitonin at very elevated levels. This study showed the complete array of the amino acid sequence of hCT and also determined that calcitonin present in a pig was different from hCT.

In contrast, both of them contained 32 amino acid peptides having a disulfide bridge near the amino terminal and also had an amino carboxy terminal. During the assessment, the gene sequence seemed to be a short arm of chromosome 11 connected directly with hCT gene CALCA (calcitonin-related polypeptide alpha) [11, 13]. Thyroid C cells are considered as the primary origin of circulating calcitonin in the human body; however, other organs such as central nervous system (CNS), lungs, and thymus also showed some calcitonin like immunoreactivity [14]. It is important to note that the patients who underwent thyroidectomy had come up with extrathyroidal excretion of calcitonin due to calcitonin-like immunoreactivity. As a result, the calcitonin was found to be present in blood and urine samples.

#### 1.1.2. Calcitonin Gene-Related Peptide

Various group research deliberately discovered the exon and silent introns and their cleavage during the maturation process in the array of eukaryotic genes [15]. Alternative splicing was demonstrated by various studies to understand the mechanism of posttranscriptional regulation. Rossenfeld et al. [16] carried out a study on medullary thyroid carcinoma line produced by the calcitonin. According to findings, few of the tumors in the process of serial transplantation altered their morphology that could be the reason of the decreased amount of calcitonin, whereas calcitonin gene CALCA has 2 alternative mRNAs, known as (i) calcitonin and (ii) calcitonin gene-related peptide. The function of these alternative species was to decrease calcitonin in cell lines instead of producing CGRP. The processing of CALCA gene involves specificity of tissues under normal physiological conditions. The splicing process occurs in neuronal tissue resulting in the generation of mRNA that further encodes the precursor of neuropeptide calcitonin gene-related peptide. However, the mRNA present in the thyroid gland C cells can be transcribed from calcitonin gene, which is also a precursor of peptide hormone calcitonin [17]. Later, it was identified that both humans and rodents possessed another different gene known to encode CGRP [18, 19] and was named as *β*CGRP (calcitonin gene-related peptide beta).

On the other hand, former gene (*α*CGRP) was named after the CALCA-encoded product. The *β*CGRP is considered as the only mature transcript of CALCB gene. A 116-amino acid precursor of the calcitonin, procalcitonin, is an additional constituent of CALCA gene. The normal physiologic process starts under specific conditions, which involve an expression of CALCA mRNA and convert it into a form that encodes procalcitonin. It is mostly restrained to the C cells of the thyroid gland, where mature calcitonin is quickly produced by cleavage of the precursor protein. In these conditions, the level of procalcitonin is decreased in the body [20]. The circulating levels of procalcitonin are rapidly increased due to involvement of infection induced by bacteria, and the organs and tissues start expressing CALCA gene.

Numerous studies conclude the fact that stimulation of procalcitonin occurs directly by bacterial involvement or indirectly by mediators. However, the procalcitonin produced due to bacterial infection is not known mechanistically. During bacterial infection, the production of procalcitonin is rapidly increased in the circulation, while during the other infections like a viral infection, the production of calcitonin is not seen. Hence, clinically, procalcitonin is used to elucidate the threats of producing septic shock; thus, it can be used as a biomarker [21]. A clinical trial of the meta-analysis involved 583 persons in which the procalcitonin was used as a biomarker to assess the infection of bones and joints such as osteomyelitis [22]. In recent studies, it was reported that osteoclast differentiation was inhibited by procalcitonin in cultures prepared from bone marrow, and surprisingly, the calcitonin receptor did not play any role to induce this process [23].

#### 1.1.3. Amylin

The pancreatic islets of Langerhans contain amyloid in the patients of type 2 diabetes (T2D). Amylin or islet amyloid polypeptide can be purified from pancreatic deposits of T2D person [24, 25]. Amylin is recently discovered as a 37 amino acid peptide that is identical to calcitonin gene-related peptides. In T2D patients, amylin can be aggregated to form amyloid or amylin fibrils [26]. In patients with T2D, the aggregated role of the amylin is still not clear, while there is evidence of their contribution to cell necrosis and the damage of islets of *β*-cells. The latest studies on eukaryotic islets transplantation showed that oligomers are the main contributing factor involved in sequence-wise loss of *β*-cells [27, 28]. The healthy pancreas contains amylin in *β*-cells, as observed shortly after the purification of amylin from the amyloid deposits. Amylin is stored in the same cellular granules in which insulin is also present, whereas the level of amylin in the granule is only ∼1-2% of insulin's concentration. Hyperglycemia stimulates the secretion of amylin although hypoglycemia decreases the secretion of amylin, while amylin is cosecreted with insulin [29, 30]. Amylin stimulates the glycogen breakdown in skeletal muscle, as the amylin shows reverse behavior to that of insulin in case of glucose metabolism [31]. Various body organs are known for the production of amylin such as gastrointestinal tract (GIT) and CNS apart from the pancreatic *β*-cells [32]. The average level of amylin is 5–10 pmol/L in a normal healthy person that is increased to 10–20 pmol/L after eating [33]. The research studies also reported that amylin level was found to be very high in obese and T2D models of humans and animals [34–37].

#### 1.1.4. Adrenomedullin

Adrenomedullin (ADM) was first discovered in pheochromocytoma in human peptide hormone by Kitamura et al. during a process of searching those features that were involved in increasing level of cAMP in platelets [38]. The peptide was highly expressed in normal adrenal medulla, and it was also suggested that adrenomedullin is the latest hormone participant in the regulation of blood pressure, as it produces a long-term hypotensive effect. Furthermore, studies were carried out. Their results reflected the central role of adrenomedullin as a vasodilator due to its effect on the cardiovascular system [39, 40]. Other groups also reported that the expression of adrenomedullin induces various pathological processes such as sepsis and kidney failure. On the contrary, pregnancy also increases the level of adrenomedullin [39]. There are various processes of adrenomedullin, which are reported to generate pathological and physiological changes in the body, such as oxidative stress and excretion of hormones, proliferation, and differentiation [41].

#### 1.1.5. Intermedin (Adrenomedullin 2)

Mammals contain only one gene of adrenomedullin family although fishes contain 5 different kinds of genes that encode five adrenomedullin peptides [42]. Intermedin or adrenomedullin 2 name was given to mammalian gene ADM2, which encodes a peptide with high similarity to adrenomedullin [42, 43]. Intermedin was discovered in human, rat, and mouse, while intermedin mRNA was present in the kidney, submaxillary gland, stomach, ovary, pancreas, and lymphoid tissues but absent in the adrenal medulla of mice. In mice, intermedin intravenous injection reduced arterial pressure efficiently as compared to adrenomedullin. The studies have reported that intermedin is expressed in GIT and pituitary gland in rat, reporting that blood pressure is reduced by intraperitoneal administration of intermedin in hypertensive rats [43, 44].

#### 1.1.6. Calcitonin Receptor- (CTR-) Stimulating Peptide (CRSP)

The isolation of CRSP was exhibited from the porcine brain [45]. There are two added peptides separated from porcine, which showed similarity to CRSP; that is why, these are linked with CRSP-1-3 family, with high affinity towards CGRP [46]. CRSP-1 is primarily expressed in the CNS and thyroid gland. In an anaesthetized rat, administration of CRSP led to a reduced level of serum calcium, whereas the blood pressure of the rat was not affected by this modification. It was also reported that CRSPs were present in other mammals such as horse and cattle, whereas a human, rat, and mouse have no CRSPs [46].

### 1.2. Production and Conformation of Calcitonin Peptides

In humans, five homologous genes encode calcitonin peptides. The chromosome 11 carries genes called CALCA, CALCAB, and ADM; chromosome 12 carries ADM2, while chromosome 22 has ADM2 gene. CALCA contains six exons in which exons I–III are situated in calcitonin as well as *α*CGRP Mrna. Similarly, exon IV and V encodes calcitonin and mature *α*CGRP, respectively, while CALCA spans ∼5.6 kb [47]. The genetic engineering performed on CALCA gene must be tissue-specific, while 95% of genes undergo processing to encode *α*CGRP mRNA in neuronal tissue. However, in thyroid C cells, 99% of the main RNA transcript undergo processing to generate calcitonin mRNA [48]. The CALCAB gene and CALCA are similar in structure. However, due to variation in the sequence, *β*CGRP is the only peptide that is encoded by this gene that showed variation from *α*CGRP by three amino acids in human and one amino acid in the rat. ADM with four exons can encode adrenomedullin. The IAPP and ADM2 genes contain exons, each three, where IAPP and ADM2 gene encodes amylin and adrenomedullin 2, respectively.

The calcitonin family has the property of producing the mature peptides through the splitting of proteins and posttranslational changes. Several structural features of peptides of calcitonin family are similar; two cysteine residues are connected with disulfide bridge at the NH_2_ terminal to present a ring-like structure, where there is an *α*-helical midregion, and carboxyl terminal contains an amidated amino acid. Among all calcitonin, CGPR, and amylin, the NH_2_ and COOH terminal remain preserved, while the middle area grows in a different direction [49]. NH_2_ terminals play the most crucial part of the stimulation of receptors [50]. The removal of NH_2_ terminal produces linear peptides, comprising *α*CGRP_8–37_, amylin_8–37_, and adrenomedullin_22–52_ that exist as the antagonists of the parent molecules, which successfully bind to the receptors having no effect on their activation [51, 52].

Calcitonin is also reported in different animal species. The primary origin of calcitonin is the ultimobranchial body in all mammals and nonmammals including birds, fishes, and reptiles [14, 53]. Surprisingly, by the examination of all the species, it was found that the cysteine residues at positions 1 and 7 and the overall length of 32 amino acids remain intact. Thus, calcitonin generated from two teleost species has been made useful for clinical applications. Asu 1–7 eel calcitonin analogue, “katonin” is categorized in eel calcitonin derivative, in which hydrogen atom replaces the NH_2_- terminal amino group, and the ethylene linkage is replaced by the disulfide bond [54].

In comparison to the parent molecule, the stability of elcatonin is improved by these modifications while retaining the biological activity. The alleviation of pain and suppression of bone and joints were seen by the elcatonin trial, and trials for several indications were also performed [55]. Salman calcitonin (SCT) differs by three amino acids from eel calcitonin, and also it shows 50% of correspondence towards peptides of human. Biological potency in humans is much higher in SCT as compared to hCT and has been used as a preparation in clinical practice widely [56].

### 1.3. Receptors of Calcitonin Peptides

Calcitonin receptor belongs to the 7TM domain, also known as G-protein coupled receptor. 7TM stands for 7 transmembrane domain of class II receptors. This group can bind with regulatory peptides such as glucagon and secretin. The earliest CTR cDNA was cloned from porcine [57]. Later on, the series of cloning was done on the receptors achieved from rats and humans [58–61]. In several tissues, CTR expression was observed, not only in the kidney and nervous system cells but also in the mature osteoclasts [62]. The chromosome 7 carries the allocated pace of human CTR and CALCR gene and span 150 kb and contains 14 exons. By alternative splicing, several forms of calcitonin receptors are formed; the difference between most common ones from each other is intracellular domain 1 by having 16-amino acid sequence and even in the absence of this sequence [14]. In rodents, two isoforms were found, with a difference in extracellular domain 2 in the region of 37 amino acids. In both humans and rodents, the negative insert form is predominant. The two isoforms produced by the alternative splicing shows cell specificity and functional implementations as it may respond to both ligands of the calcitonin family and the downstream signaling mechanisms [14]. Two research groups cloned CTR-like receptors (CRLR), and its sequence was similar to that of CTR [63, 64]. In human, CRLR encodes by a gene, which contains 15 exons and the genomic DNA span over 103 kb and is situated on chromosome 2. Previously, CRLR was considered as an “orphan receptor” [65]. After that, the study conducted by McLatchie et al. [66] discovered that receptor modifying proteins (RAMPs) produce two identical molecules with CRLR as calcitonin family peptides work for specific receptors. Only 3 RAMPs are being recognized as they contain structural homology of NH_2_- terminal, *α*-helix, and COOH-terminal. RAMP1 carries six cysteine residues, while RAMP2 contains four residues in the extracellular domain. Evidence has suggested that 2–4 and 3–6 cysteines, which resides between disulfide bridges, are mandatory for the formation of a complex called CALR/RAMP complex, and this complex is necessary for the safety and stability of the molecule [67].

**Figure 1:**
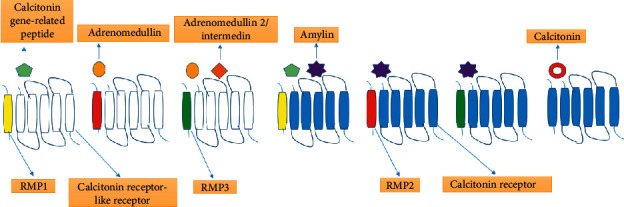
Classification and composition of human calcitonin family receptors showing various receptors (labeled below the figure) and ligands (labeled above the figure).

When CRLR dimerizes with RAMP1, a highly specific receptor for CGRP is formed. Similarly, when CRLR dimerizes with RAMP2 and RAMP3, receptor having affinity with an adrenomedullin receptor is produced [68]. Additionally, this CRLR/RAMP complex can behave as a receptor for intermedin, which in result, indiscriminately interacts with one of the three RAMPs or CRLR dimers [43]. Additionally, it was also found that RAMPs can bind with and stimulate CTR [69–71]. When CTR binds with calcitonin, specific amylin receptors are formed from a complex of CRLR and three RAMPs. Thus, it is found that calcitonin peptides use CTR, RAMP1-3, and CRLR as the receptor, and accordingly, the naming system has been introduced (Figure 1) [70–72]. The studies of the pharmacological effects of binding ability and the receptors of calcitonin peptides showed that every receptor binds with excellent efficacy to calcitonin family peptides; on the contrary, the other members of the family interact with lower affinity [49]. The interacting affinity depends on the experimental trial system [73]. The calcitonin family member's cross-reactivity with the combinations of several other receptors showed a challenge for experimental result's interpretation since the deficiency of each specific constituent could disguise itself with the other members of the peptides and receptor family's interactions. In research, GPCR regulation is a useful arena where GPCRs are usually used as a target for drugs. From various evidences, it is confirmed that 30–50% of the clinically used medicines act via approximately 80 members of this receptor family.

## 2. *In Vitro* and *In Vivo* Studies in WT Animals

The subsequent sections represent various studies in wild type (WT) and genetically modified (GM) animals as well as clinical investigations for the discussion of the biological role of calcitonin peptides in the bone [74–82].

### 2.1. Effect of Calcitonin on an Elevated Level of Blood Calcium

The observation that calcitonin lowers the amount of circulating calcium resulted in the hypothesis that its physiological function in hypercalcemia might be involved in restoring ordinary concentrations of serum calcium. In several *in vitro* studies, this hypothesis was tested in rats, many of which were parathyroidectomized (PTX) without PTH-secreting cells or thyroparathyroidectomized (TPTX) without C-cells, which secrete both PTH and calcitonin. When calcium injection or infusion directly caused hypercalcemia, the existence of thyroid gland was essential to reduce the calcium concentrations in the circulation [83, 84]. Same conclusions were observed with the IV administration of parathyroid or partially purified PTH-induced hypercalcemia, conforming its advantages in hypercalcemia [85]. A general finding in renal failure is the resistance produced due to the calcemic mechanism present in PTH and secondary hyperthyroidism. Rodriguez et al. documented the fact that the presence of thyroid gland is mandatory in suppressing the calcemic action to PTH in rats. This reduction can be seen in the PTH-induced hypercalcemia in both cases of PTH-induced hypercalcemia associated with kidney failure or diet-provoked hyperparathyroidism [86]. It was also suggested that the biological characteristics of calcitonin in bone loss protection was affected by other hormones.

The TPTX rats with decreased calcitonin were treated with PTH to produce an increased level of calcitonin, which resulted in bone damage from the proximal part of the tibia side. On the contrary, the PTX rats that had sufficient calcitonin came up with no damage to the bone, thus rendering the protective function of calcitonin, under these circumstances [87]. Based on the findings that osteopenia caused by ovariectomy (OVX) rats is involved with a reduction in the circulation of calcitonin, it was suggested that calcitonin stimulated the degradation process of bone caused by estrogen deficiency. However, this hypothesis has not been supported by experimental proof. For example, in rats, with and without thyroid gland showed a nonsignificantly different decline in femur density and calcium content [88]. The key strength of the research mentioned above is the usage of animal models that enable careful manipulation of hormone level by removing thyroids, parathyroid, and ovaries. These results confirm that the key origin of calcitonin is thyroid and shows the potential to cope up with an increased level of calcitonin due to various reasons. These studies did not illustrate response to the fundamental issue of the biological and functional role of CT; despite these apparent improvements in scientific understanding, it defines its function in a pathology. The growth of those mice that are genetically altered in subsequent years has given the potential objective for further research towards the contribution of calcitonin in stressed conditions.

### 2.2. Bone Resorption by Osteoclasts

Mature osteoclasts are created by merging of precursor hematopoietic cells and play a vital role in bone resorption. A high number of local and systemic variables regulate the differentiation and activity of osteoclasts. The interaction of macrophage colony-stimulating factor to its receptor c-FMS induces the osteoclast differentiation, which results in stimulation of expression of nuclear factor-kappa-*β* (RANK). The RANK-RANK ligand (RANKL) interaction stimulates the differentiation and activation of osteoclasts. Osteoprotegerin (OPG) is an osteoblast lineage cell-secreted decoy receptor, which interacts with RANKL and competitively inhibits RANK/RANKL interaction. Thus, the main factor in controlling osteoclast activation and bone resorption is the ratio between OPG and RANKL levels [89]. Mature osteoclasts degrade the extracellular matrix of the bone, utilizing a specific methodology that is already documented in previous studies. When mineralized bone matrix interacts with fully differentiated osteoclasts, a “sealing region” is produced that covers the resorption lacuna's enclosed area. The osteoclast membrane present in the sealing arena undergoes twisting and forms a tangled border. These borders are meant to be used for the transportation; i.e., protons, and matrix-degrading enzymes are released during the bone resorption method. Shortly after the discovery of calcitonin, it was found that the bone resorption is the vital factor for the fast decrease in calcium concentration of the circulation under the effect of calcitonin [11]. After some years, the complete detail about calcitonin family was described [90]. Furthermore, the osteoclasts exhibit the expression of CTR, RAMP1-3, and CRLR, revealing the interaction potential of the calcitonin family with these cells [91].

#### 2.2.1. Calcitonin

Calcitonin binding to osteoclasts receptor results in the loss of the ruffled boundary within minutes, which results in cell removal along with its restriction of movement and bone dissociation [92, 93]. Several different mechanisms can stimulate calcitonin activity in the osteoclasts, such as cAMP affects motility restriction and quiescent state induction. At the same time, intracellular calcium signaling mediates the removal along with disruption of the resorption process in the sealed area [94, 95]. Various studies on the mechanistic aspects of sealing area unbinding revealed about calcitonin-based modulation of Src and tyrosine kinase Pyk2 that is strongly expressed in osteoclasts and located primarily in the sealing area [96, 97]. When mature osteoclasts of the mouse were administered with sCT, it did not affect the amount of osteoclast cells in culture; however, the ability of bone resorption by the pretreated cells was reduced [98–101]. The sCT-treated cells exhibited pits of smaller size as compared to control cells, indicating a prolonged suppressive impact of sCT on the motility of osteoclast.

#### 2.2.2. Calcitonin Gene-Related Peptide

Resembling to the calcitonin impacts, hypocalcemia was induced by injecting CGRP to rabbits and rats [102, 103]. Multiple studies discovered that CGRP due to its inhibitory effect show inactivation of osteoclast; thus, bone resorption was also seen. However, the efficacy of CGRP is too low as compared to calcitonin. The cells were cultured from the bone marrow of mouse under the effect of M-CSF and RANKL; CGRP at 0.1 nM or higher concentration reduced the area of resorption pits, while 10 nM CGRP was needed to inhibit TRAP-positive cell development. According to these findings, osteoclast activity is more strongly inhibited compared to bone resorption under the effect of CGRP [104]. CGRP also adversely affected the production of TRAP-positive mono- and binuclear cells. After treatment with CGRP, the levels of osteoclasts were reduced in the cultures of the human bone marrow [105]. This experiment revealed that CGRP binds to osteoclast precursors, leading to the regulation of osteoclast production.

In animal models, the impact of CGRP on bone resorption was evaluated. OVX is a standard process conducted to produce osteoporosis in the animals with a deficiency of estrogen. In a study, the administration of CGRP to OVX rats hindered the bone resorption [106]. CGRP is less efficient in suppressing bone resorption than sCT although it has been tested at a concentration 500 times greater than sCT. This reduced efficacy indicates that this may not be an activity due to the action of CGRP on its receptors; rather, it could be a nonspecific effect of CGRP [106].

The literature study has proposed a function for CGRP in defending bone from adverse effects of bone implantation. After joint arthroplasty, aseptic loosening may happen as a consequence of adhering particles produced from the implant leading to local bone resorption. The osteolysis caused by wear particles is a critical reason for the failure of the implant. The existence of CGRP in the skeleton and nerve fiber periphery of periprosthetic osteolysis locations emphasizes to study whether CGRP exhibits protective effect via suppression of osteolysis by osteoclasts [107, 108]. An *in vitro* study was conducted on osteoblasts and osteoblast-like cell line MG-63 to study the influence of CGRP on the catabolism induced by ultra-huge molecular weight polyethene (UHMWPE) particles [109, 110]. UHMWPE particles caused RANKL expression but inhibited OPG expression in both kinds of cells; on the contrary, CGRP lowered RANKL level produced by UHMWPE, indicating suppression bone resorption under the effect of these particles.

#### 2.2.3. Role of Amylin

Early trials discovered that injection of amylin caused hypocalcemia [111–115]. Another study reported that amylin hindered the formation of TRAP-positive cells [99]. According to another study, amylin inhibits cell fusion through activated signal-regulated protein kinase 1/2 (ERL1/2) [116]. Additionally, amylin contributes to bone disruption by mature osteoclast [99]. In mouse neonatal calvarial organ culture scheme, amylin enhanced concentrations of cAMP and decreased the PTH-stimulated resorption [52, 117]. The bone disruption in a fetal mouse can be overturned by amylin; however, its efficacy was comparable to the efficacy of CGRP; when compared to hCT, the efficacy decreases up to 60 times [118]. The administration of amylin either locally for five days or systematically for one month results in a 60–70 percent decrease in bone resorption indices [107, 119]. Another study involved the administration of amylin in estrogen-deficient rats for 30 days. Amylin decreased urinary excretion of deoxypyridinoline in these experimental animals and decreased the trabecular bone loss although it did not affect cortical bone indices [120].

#### 2.2.4. Role of Adrenomedullin

Despite the adrenomedullin receptor's presence on osteoclasts and the capacity of adrenomedullin to cause cAMP formation in these cells, studies have continuously demonstrated that the osteoclast differentiation is not affected by adrenomedullin. This phenomenon has been studied in the cultures having 1, 25(OH)_2_D_3_ or M-CSF and RANKL to produce osteoclasts in bone marrow cultures [66, 121, 122]. This is unlike to all the other calcitonin family members. However, in some pathological circumstances that cause bone disruption, adrenomedullin acts differentially by modulating the inflammatory environment to hinder bone disruption. In an *in vitro* model, the treatment of rheumatoid synovial fibroblasts was provided by giving IL-1*β* and TNF-*α* proinflammatory factors and cultured along with mononuclear peripheral blood cells. In another study, osteoclast development was suppressed in the cells treated with adrenomedullin, via expression of RANKL and OPG [123].

#### 2.2.5. Intermedin

In osteoclast, intermedin activity is distinctive from that of adrenomedullin. However, its activity is equivalent to amylin, calcitonin, and CGRP. Intermedin is the potential inhibitor of multinucleated osteoclast development, which is regulated by M-CSF and RANKL. In this study, cAMP mediated the activity of intermedin [122, 124]. Current studies have indicated that the MC3T3 osteoblastic cells treatment with intermedin may hinder the formation of osteoclasts due to increase in OPG expression and decrease in RANKL and M-CSF expression [125].

#### 2.2.6. Comparative Studies

There are various studies on the comparison of the action of calcitonin peptides on the development and activity of osteoclasts. The formation of osteoclasts is inhibited by amylin, sCT, and human CGRP in the 1, 25(OH)_2_D_3_-containing culture of the mouse bone marrow [99]. At a level of 0.1 pmol/L and above, sCT suppressed osteoclast differentiation, while at a low level of 1 nmol/L or more, the activity of amylin or CGRP is decreased [99]. Granholm et al. [101] reported the similar findings, i.e., inhibition of osteoclast formation in the bone marrow and spleen of the mouse under the effect of Sct [101]. The formation of osteoclast induced by calcitonin was tested in the M-CSF- or M-CSF-RANKL-treated bone marrow of mouse to provoke osteoclast differentiation [91]. M-CSF-treated bone marrow cells showed the expression of mRNA and proteins of CRLR and RAMP1-3, whereas cell treatment with adrenomedullin, amylin, intermedin, or CGRP promoted cAMP formation [91]. Furthermore, the CTR expression was provoked by the addition of RANKL, and CTR expression was accompanied with the cell's responsiveness to sCT. Interestingly, amylin showed its action in M-CSF-treated bone marrow cells without exhibiting expression of CTR. It shows that amylin acts through receptors other than CTR/RAMP1-3 in these cells.

The influence of calcitonin peptides on bone resorption stimulated by PTH was measured in organ cultures by determining ^45^Ca release from mouse calvaria [122]. Comparison of peptide's inhibitory impacts on bone disruption depicted that sCT was the most potent moiety having IC50 = 3 pmol/L. However, CGRP and amylin had IC50 at 10–30 nmol/L and intermedin at 300 nmol/L, and adrenomedullin had no impact [122]. Furthermore, microscopy and image analysis techniques are useful in the comparison of amylin, calcitonin, and CGRP effects on the osteoclast. Osteoclast motility was decreased by CGRP and amylin, yet the calcitonin suppressed both the motility level and osteoclast withdrawal. The study showed that the motility of cell is mediated by the cAMP signaling pathway. As a conclusion, cAMP pathway is activated by amylin and CGRP, while calcitonin level influences osteoclasts, likely via calcium level change in cells. This phenomenon might be responsible for the greater potency of calcitonin in the suppression of bone dissociation [126].

### 2.3. Role of Osteoblastic Differentiation in Bone Matrix Production

The mononuclear bone-forming cells, named as osteoblasts, undergo differentiation from the mesenchymal stem cells of bone marrow. Under the effect of an organized signaling pathway, preosteoblasts are evolved into mature osteoblasts that generate bone matrix and then mineralize it. After bone formation, the osteoblast can either undergo apoptosis or be attached in the matrix of bone, leading to their differentiation into osteocytes [127–132].

#### 2.3.1. Calcitonin

Several studies documented the impact of calcitonin on the division of osteoblast and development of bone. A latest study depicted that calcitonin treatment was used for bone development and division of osteoblast. Even after completion of bone formation process, it hinders the bone development process [133]. The succeeding research discovered a fast stimulating impact of calcitonin on the multiplication of osteoblast [134, 135]. Calcitonin, on the other side, showed no activity on the osteoblastic proliferation in rats and did not change indices of bone development in adult mice that received a local injection of calcitonin [51, 107].

An experimental trial proved the absence of CTR expression in the osteoblasts [136]. Due to a high concentration of peptide used in this study, calcitonin activity could be due to its interaction with receptors other than CTR. Furthermore, due to possible presence of other bone cells in the primary osteoblast culture, its indirect impact on osteoblast was evaluated. Later researches in the animal models reported that calcitonin regulates bone formation, but the action discussed above was generated by osteoclasts and osteocytes rather than a direct impact on osteoblasts.

#### 2.3.2. Calcitonin Gene-Related Peptide

In contrast to calcitonin, CGRP has a beneficial impact on osteoblasts in vitro, but not in vivo. It has been demonstrated that CGRP specifically binds to the rat's calvaria cells [137]. After treatment with CGRP treatment, the cAMP level is elevated in the cell lines of UMR 106-01 rat with osteosarcoma, osteoblastoma, and primary osteoblast cultures [138–140]. CGRP enhanced cAMP concentrations in the culture of calvarias of rat, chicken, and mouse, while calcitonin had no impact on these cells [141]. CGRP therapy improved the level of calcium in UMR 106-01 osteosarcoma cells and in cell lines of human osteoblast viz. MG-63 and OHS-4. Here, it is worthy to mention that intracellular calcium is a second messenger in CGRP signaling [142–144]. CGRP induced primary osteoblast proliferation; however, its efficacy was lower than that of amylin [145]. Also, CGRP antagonist CGRP_8–37_ [146] did not inhibit the proliferative effect of CGRP. This has made it possible for the action of CGRP and amylin on osteoblast division through the same receptor that possesses a higher efficacy for amylin [145]. *α*CGRP was also found to promote the differentiation of osteoblast in cultured bone marrow cells of rat, while *β*CGRP showed no osteogenic type activity in this experimental system [147]. Thus, CGRP also hinders the apoptosis of osteoblast-like cells via Wnt/*β*-catenin-dependent signaling pathway and enhances BMP-2 activation leading to an increase in differentiation [148, 149]. According to a finding, CGRP provokes osteoblast precursor differentiation [140, 150]. Latest studies have shown that CGRP caused differentiation in bone marrow stromal cells (BMSCs), which resulted in the mineralization of osteoblast in healthy or OVX rats [104, 151]. CGRP effect on BOMSC differentiation likely takes place through Wnt/-*β*-catenin pathway induction [152–154].

#### 2.3.3. Role of Amylin

Various studies have reported the role of amylin in the development of cAMP in the cell lines of primary osteoblastic cells, fetal osteoblastic cells of rats, as well as primary osteoblast cell of human, showing bone as a possible target of amylin [107, 118, 155]. The investigations on the signaling pathways involved in the amylin-mediated proliferation showed that G_i_ proteins activate ERK1/2 phosphorylation that alternatively induces mitogenicity under the effect of amylin in the osteoblast-like cells of rats since an inhibitor PD-98059 suppresses the proliferative effect of amylin. It was a surprise that the amylin-induced proliferation in osteoblasts needed the IGF-1 receptors in spite of the absence of any interaction between amylin and IGF-1 receptors [156].

According to various studies on the function of amylin, the NH_2_- terminal octapeptide fragment amylin_1-8_ activated the proliferation of primary rat osteoblast [52, 157]. The presence of small chain amylin that exerts mimicking effect on bones offers a chance for the development of amylin-based therapeutic moieties. Stable analogues of amylin 1–8 are undergrowth for future use in osteoporosis [158, 159]. Furthermore, amylin works *in vivo* to promote the development of bones. When given locally daily for five days to mouse calvariae, amylin resulted in a fourfold increase in the osteoblast activity [107].

#### 2.3.4. Role of Adrenomedullin

Analogous to amylin, adrenomedullin plays a significant role in the proliferation either in rat or human osteoblast and in the culture of ex vivo neonatal mouse calvaria [160–164]. Adrenomedullin is expressed mostly in osteoblasts and shows activity via a paracrine/autocrine mechanism [165]. Dissimilar to other peptides of calcitonin family, adrenomedullin shows uncertain impacts on osteoblast cAMP levels showing that adrenomedullin in these cells activates ERK1/2 and voltage-dependent calcium channels [166, 167]. Additionally, adrenomedullin exhibited no direct binding to IGF-1R, and like amylin, its effect of proliferation in osteoblastic cells shows dependency on IGF-1R [156]. Adrenomedullin is considered as a survival factor. It suppresses the apoptosis process in osteoblast, probably via the signaling pathway ERK1/2, activation of CREB, and Wnt pathway [167, 168].

#### 2.3.5. Role of Intermedin

In terms of its effects on osteoblasts and bone formation, intermedin is the CT family's least studied peptide. In vitro research has shown that intermedin does not influence the proliferation or differentiation of MC3T3 osteoblast-like cells. Yet, it appears to suppress dexamethasone and apoptosis-induced serum starvation, indicating its widespread beneficial impact in these cells [125]. To comprehend intermedin action in cells along with its prospective action on the development of bone, still advanced studies are required for better understanding.

### 2.4. Function of Osteocytes in Bone

Osteocytes are present in lacunae within the mineralized bone tissue. Osteocytes interact with each other via dendritic processes. Osteocytes have significant contribution in the regulation of bone turnover and mineral metabolism. The latest studies have discovered that osteocytes create osteoclast-like characteristics and undergo osteolysis along with the removal of bone matrix into their extracellular space [169]. Osteocytes express genes, known as osteoclastic markers such as cathepsin K and TRAP [169, 170]. Osteocytes produce sclerostin, which is a vital glycoprotein and encoded by the SOST gene. Sclerostin interacts with coreceptors LRP4 chaperone and LRP5/6 and suppresses the signaling pathway of Wnt/catenin [171]. Sclerostin hinders the development of bone in transgenic animal and promotes bone resorption by affecting osteoclast precursors and RANKL/OPG pathway. Monoclonal antibody-targeting sclerostin in humans is an antiosteoporotic drug, which is in final stage of its development [172–174].

### 2.5. Discussion of In Vitro and In Vivo Studies in WT Animals

**Figure 2:**
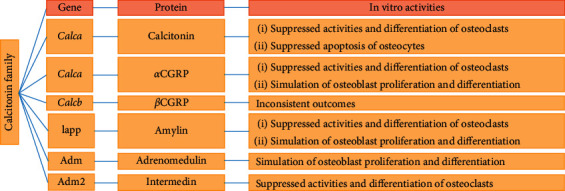
Calcitonin genes family and their respective *in vitro* effects on bone cells. CGRP, calcitonin gene-related peptide.

In the context of bone remodeling, Figure 2 shows a summary of calcitonin family peptide activity in bone cells. The peptide activities differ in the efficacy and specificity, and it is confirmed that the calcitonin family peptides in the bone produce an overall beneficial impact. Normal concentrations of calcitonin-peptides in human serum are in picomole per liter range. Abovementioned studies used these peptides in a concentration range from picomole per liter to micromole per liter. Except CGRP, which can hit an increased native amount of microenvironment of bone, the experiment conducted on calcitonin family peptides at elevated amount of drug needs to be carefully monitored. Moreover, the sCT is broadly utilized during the experimental trial conducted on mouse, rat, and human systems that surely hinder the possible outcomes of pharmacology-related outcomes, instead of physiological outcomes. Furthermore, though the physiological relevance may be doubtful, the assessment of sCT and amylin at elevated levels is clinically essential during the use of sCT and the human amylin analogues such as pramlintide, especially in patients suffering from diabetes [175–179]. Belowmentioned discussion reveals the importance of genetically modified animals in improving the knowledge about bone activity (in context of physiology) of calcitonin peptides.

## 3. *In Vitro* and *In Vivo* Studies on GM Animals

### 3.1. Physiological Function of Calcitonin

Since the invention of calcitonin over 50 years ago [6], its inhibitory effect on bone resorption has been thoroughly investigated. Thus, it is astonishing that calcitonin role in context of physiology is vague. Calcium metabolism and mineral density of bone are not affected in patients with medullary thyroid carcinoma with a chronically increased level of endogenous calcitonin or in thyroidectomized individuals with undetectable circulating calcitonin [180, 181]. As there is no pathological effect of an increased or decreased excretion of calcitonin, a study also recommended that calcitonin shows no biological or functional impact on mammals. According to a study on the vestigial nature of calcitonin, it was found that calcitonin is essential for fish survival, but it does not play any role in the mammalian hemostasis of calcium or bone disruption [182–184]. Unlike these dubious opinions, the present harmony is that, under calcium stress conditions, calcitonin plays a vital role in protecting the skeleton.

### 3.2. Role of Calcitonin, Calcitonin Gene-Related Peptide, and Calcitonin Receptor in GM Mice

The lab research conducted in GM mice found that calcitonin profoundly suppresses the bone disruption process, and hence, calcitonin-deficient mice (CDM) were supposed to reduce bone mass, as compared to control breed of wild-type (WT) mice, due to an enhanced resorption of bone. Nevertheless, the bone phenotype of mice having a deficiency of calcitonin was completely different from what was expected. Hoff et al. introduced a mouse breed with decreased level of calcitonin and *α*CGRP due to absence of the required DNA sequences and were named as Calca knockout (KO) or CT/*α*CGRP^−/−^ [185]. The mice showed no effect on their growth, and their calcium concentrations were normal. Surprisingly, due to enhanced bone formation, the Calca KO mice had a considerably higher volume of trabecular bone mass. Calca KO mice showed a low sensitivity to OVX, retaining their bone mass; however, bone mass of WT mice was reduced to three times after two months of OVX. The Calca KO mice also revealed that the osteoblast can be considered as an important target of calcitonin/*α*CGRP in bone and that calcitonin/*α*CGRP hinders bone development. Nonetheless, mice did not contain both calcitonin and *α*CGRP, and this phenotype could not be attributed to any peptide [185]. Following the publication of original research, the same group defined the phenotype of *α*CGRP KO mice having intact calcitonin [186]. The calcitonin level of *α*CGRP KO mice was normal, and in contrast to Calca KO, the rates of bone development were decreased leading to osteopenia formation. Thereby, calcitonin deficiency resulted in the elevated bone mass phenotype of the Calca KO of mice. At the same time, *α*CGRP seems to be involved in the enhancement of the bone development. Another study on Calca KO mice proved the development of the morphological characteristics of bone with age. The enhanced bone formation in animals 12 and 18 months of age was accompanied with a similar rise in the disruption of bone, with an increased rate of bone turnover in 20% of the Calca KO mice. These findings suggested an inhibitory effect of calcitonin on the bone phenotype of the older KO mice [187]. Unlike age-dependent development of phenotype in Calca KO mice, *α*CGRP KO exhibited osteopenia phenotype at all the analyzed ages [187]. The presence of an integral Calcb gene encoding *β*CGRP confounded the phenotype analysis of the Calca KO and *α*CGRP KO mice. Nonetheless, there was no difference in the bone phenotypes of Calcb KO mice and control WT mice. Thus, it can be concluded that *β*CGRP has no significant contribution in the regulation of bone remodeling [188]. To study the adaptive response to mechanical loading (ML), ML to ulna could be adopted as a model, which was used later on in other studies for testing the role of CGRP adaptive response to ML. WT mice showed the activation of periosteal mineralization under the effect of ML; however, it was not seen in *α*CGRP KO mice.

### 3.3. Inferences and Qualms regarding Calcitonin, Calcitonin Gene-Related Peptide, and Calcitonin Receptor

Skeletal biology has significant association with calcitonin and its receptors. Overall, the Calca KO mice and Calcr KO mice have been regularly observed to have elevated bone mass due to enhanced development of bone although Calcr expression is not observed in the osteoblasts. A later study resolved this contradiction successfully. According to the finding, there was an increased bone formation in the skeletal phenotype of an osteoclast-related Calcr KO, like Calcr KO [189–191]. In addition, calcitonin enhanced the quantity of sclerostin, secreted from osteocytes. Thus, calcitonin affects osteoblasts indirectly, likely through two kinds of bone cells, which undergo Calcr expression, named as osteoclasts and osteocytes. The skeletal morphology of mouse lacking calcitonin requires further studies. Calcitonin activity has so far been concluded from the skeletal phenotypical characteristics of mice lacking both calcitonin and *α*CGRP and mice missed in *α*CGRP alone. However, there is no direct assessment of a mouse lacking calcitonin. An additional significant issue was the signaling of amylin in the Calcr KO mouse. As the identified amylin receptors include CTR, the investigation of the Calcr KO phenotype in this mouse model should highlight the fundamental impact on amylin signals. Calcitonin's pharmacological study in the specific bone cells of genetically modified animal has determined the physiological role of calcitonin in hindering bone development. A same kind of issue has been recognized in studies of PTH action in bone since PTH stimulates the formulation of bone [192].

### 3.4. Effect of Calcitonin and Its Receptor in Elevated Level of Calcium

To study the role of CTR in maintaining calcium homeostasis in hypercalcemia, the CTR KO-mouse model outlined above [191] was used. When 1, 25(OH)_2_D_3_ induced hypercalcemia, the calcium level in the CTR KO mice was significantly higher, indicating that CTR is essential in calcium stress [191]. The similar animal model was used to explore osteoclast-expressed CTR contribution to prevent an increase in the level of calcium [193]. In three mice strains, the researchers compared its action to hypercalcemia: CTR removal in WT, global CTR KO, and a Cre/loxP mouse models in osteoclast bone. With high serum concentrations of calcium, the KO strains showed the same responses to the increased calcium level. These findings indicate that calcitonin predominantly protects against hypercalcemia by inhibiting osteoclast activity.

### 3.5. Role of Calcitonin in Lactation

The function of calcitonin and CTR during lactation was studied by using transgenic animal models. Lactation is a biological condition that is accompanied with fast mineralization to provide the milk with calcium. The study described a 50% decrease in mineral contents of spine bone in the Calca KO model, and the WT controls showed a decrease of 23.6% only [194]. The spine bone minerals were normalized in the WT mice after thirteen days of feeding, while it might surpass 18 days to achieve the values in the Calca KO mice. In addition, the effect of sCT and CGRP in the double-KO mouse model was assessed. The administration of sCT brought bone parameters back to normal, while there was no effect of CGRP. It showed that calcitonin significantly contributes to calcium homeostasis and maternal structure normalization after the weaning period [194].

The studies on global CTR KO mice have also suggested that calcitonin plays its role in defending the skeletal state from extreme resorption of bone during lactation [195]. The resorbing capacity of osteoclasts was the same among both CTR KO and the WT animals. Nonetheless, the CTR KO mice have been found to have enhanced expression of several genes such as Catk and Mmp13 in tibiae. The researchers demonstrated previously that osteolytic genes were expressed in osteocytes during calcitonin deficiency. In addition, CTR KO mice showed a larger osteocyte lacunar area compared to that of WT, indicating a functional role for calcitonin in the suppression of osteocytic osteolysis, resulting in the protection of maternal bones during the span of lactation [195].

### 3.6. Role of Amylin in GM Mice

Dacquin et al. investigated the morphological characteristics of bones in amylin-deficient mice [116]. In this experimental model, the deficiency of amylin had no impact on regulating ingestion of food, body weight, or glucose metabolism. After an experiment of 24 weeks, osteoporosis was noted in both male and female mice with amylin KO features. Both amylin KO and WT mice had similar number of osteoblasts. However, osteoclasts number collagen degradation products were higher in the amylin KO mice, showing that a swift bone resorption resulted in the osteoporotic phenotype [116]. Bone phenotypes of young and adult male and female amylin KO mice were further compared. Trabecular thickness of amylin KO mice at an age of six weeks and femoral length at an age of seven months was increased, while female mice and WT control mice had similar measures [196]. In short, different experiments showed similar impact of amylin on osteoclasts and bone resorption. Osteoporosis was observed in amylin deficiency. In addition, osteoclast differentiation was suppressed by amylin.

### 3.7. Role of Adrenomedullin, CTR-Like Receptor, and Ramp1-3 in GM Mice

Some beneficial findings were obtained from the mice strains deprived of capability to produce adrenomedullin, Calcrl, Ramp1, Ramp2, and Ramp3 [41, 197]. Genetic deprivation in either adrenomedullin, Calcrl, or Ramp2 [198–200], is embryonically fatal in midgestation due to cardiovascular defects and hyperproliferative lymph vasculature. However, other strains such as Ramp1 KO and Ramp3 KO exhibit excellent viability [199, 201, 202]. These results show that RAMP1 to 3 could not compensate for the damage of RAMP2 despite the structural resemblance between the three RAMPS. A conditional KO mouse model was developed by excising adrenomedullin gene through doxycycline-based Cre/Lox and utilized this model for studying the skeletal effects of adrenomedullin deficiency [203]. Due to certain etiologies, the concentrations of circulating adrenomedullin were decreased to half after treating with doxycycline. Compared with WT control, adrenomedullin-deficient mice exhibited an enhanced bone mass and density as determined in femora. In contrast to these unexpected outcomes, previous experiments with in vitro and local administration of adrenomedullin had an direct positive impact on bone formation, leading to enhanced bone mass and density in the mice lacking adrenomedullin [66, 104, 165]. Ghrelin and CGRP are two peptides that could be involved in such indirect actions and induce bone development. These findings are confirmed by the latest knowledge that KO mice became obese after doxycycline administration [203]. Moreover, the adrenomedullin inhibitor, named as the small molecules 16311, did not affect control femora, rather exerted a protective effect on OVX mice, indicating adrenomedullin effect in facilitating bone loss due to OVX [203].

## 4. Clinical Studies

Calcitonin inhibits osteoblast-induced bone resorption. Since bone mass is not affected by the absence or presence of calcitonin, it can be used to suppress turnover, by keeping bone mass constant [204–206].

### 4.1. Administration of Salmon Calcitonin (sCT)

sCT potency is higher than that of hCT; thus, it is the most commonly used calcitonin peptide among various calcitonin preparations used in clinical practice. Thus, it is widely used in clinics. sCT is considered safe and produces antibodies in patients; it is thought to have negligible undesired effects [207]. sCT was presented to market in 1974, followed by its approval by the FDA against postmenopausal osteoporosis, hypercalcemia, and Paget's illness [56, 208]. Initially, sCT was available commercially as an intramuscular or subcutaneous formulation. There were benign, yet lethal effects of calcitonin injections, but its parenteral therapy was long-lasting and uncomfortable [209]. Calcitonin formulation as a nasal spray has been applied in various studies. The adverse effects of nasal spray are its poor bioavailability as compared to its parenteral administration [210]. The recent trial of sCT as drug development is an oral preparation that enhances bioavailability and compliance [211]. The oral formulation contains sCT coupled to its delivery agent through weakly and noncovalent forces, which enhances the ability of sCT to pass through the gastrointestinal epithelium and defend it against the metabolizing enzymes [212].

### 4.2. Antiresorptive Effect of Salmon Calcitonin

An important treatment used for bone resorption was sCT. Based on the intranasal or subcutaneous application of sCT, various studies have shown an enhanced bone density, decreased fractures of vertebrae, and decreased risk of hip fracture [56, 213–215]. In contrast, others have not discovered any bone effects [216]. Another study showed the action of nasal spray on the likeliness of vertebrae fractures in postmenopausal osteoporotic females [217]. The results showed that the risk of vertebral fractures was significantly lowered by a daily dose of calcitonin 200 IU. Later on, the findings were challenged mainly because of the elevated dropout rate (59 percent) that compromised the observations since the dose of 200 IU/day showed its efficient effects, but a more significant higher dose found ineffective [218]. A later study combined a noninvasive MRI technology with iliac crest bone biopsies for the determination of sCT effect administered through nasal route on the trabecular microarchitecture of skeleton [219]. This placebo-controlled trial of 2 years on 91 postmenopausal osteoporotic females recommended a therapeutic advantage of sCT in keeping trabecular microarchitecture at a specific skeletal region. A latest study assessed the therapeutic potential of orally administered calcitonin in postmenopausal osteoporotic females [220]. Treatment group showed an increase in the mineral density of vertebral, neck, femur, and hip bone. Due to reduced efficacy in the prevention of fractures, the formation of an oral preparation of calcitonin has been suspended [220, 221]. In initial years, postmenopausal osteoporosis of females was treated using sCT. However, sCT is no more used clinically due to studies showing association between sCT use and cancer development and the development of new drugs such as bisphosphonates for the treatment of osteoporosis [208].

### 4.3. Use of Salmon Calcitonin in Bone-Related Paget's Disease

Paget's disease of bone was initially treated by using calcitonin, first ever suppressor of osteoclasts [222]. According to another study, SCT inhibited bone turnover resulting in the relief from the pain and improved bone features [56]. However, the repeated use of calcitonin resulted in resistance against it after a slight recovery. Bisphosphonates are found as a better alternative to manage Paget's disease similar to the treatment of osteoporosis. The European Medicines Agency suggests the use of sCT for short use, using its minimum effective dose [223].

### 4.4. Use of Salmon Calcitonin in Osteoarthritis

Osteoarthritis is also treated by using sCT by inhibiting bone turnover, controlling pain, and secreting cartilage proteins [56]. Several studies have shown the action of calcitonin against chondrocytes. Calcitonin caused cAMP formation in chondrocytes in an animal model, inhibited type II collagen degradation, and enhanced metalloproteinase matrix activity [224]. Type II degradation of collagen is attenuated by calcitonin in vivo [225–227]. Furthermore, the researchers produced an animal model of osteoarthritis disease with a large value of erosion index, while transgenic mice overexpressing sCT had a reduced index of erosion than WT mice [228]. CTR expression is required for a direct effect of calcitonin in chondrocytes, as shown in several studies [229].

## 5. Conclusion

**Figure 3:**
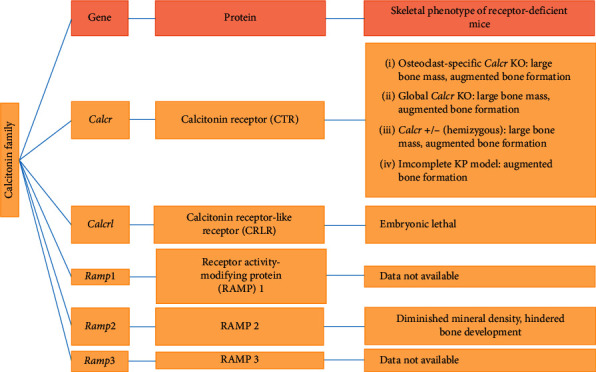
Characteristic properties of the skeletal phenotype of knockout mice.

Calcitonin peptides and their receptors have a diverse role in physiology (Figure 3), including its impact on various cells, tissues, and model animal's phenotype, showing that calcitonin affects skeletal physiology. Out of various challenges of calcitonin effect on bone activities, the most critical issue deals with the difference in its therapeutic effects in bones in vitro and in vivo. Other discrepancy is related to the genetic, physiological, and pharmacokinetic variation among different species. Osteoclast is the basic target of calcitonin-peptides in bone. Accordingly, pharmacological outcomes vary with age, health status, and environment. Although more effective pharmacological agents have replaced calcitonin use in accelerated bone turnover, there is still a promising attention in the calcitonin peptides and their effects on skeleton.

## Data Availability

All the data have already been added to the article.
